# Identification and epidemiological characterization of Type-2 diabetes sub-population using an unsupervised machine learning approach

**DOI:** 10.1038/s41387-022-00206-2

**Published:** 2022-05-27

**Authors:** Saptarshi Bej, Jit Sarkar, Saikat Biswas, Pabitra Mitra, Partha Chakrabarti, Olaf Wolkenhauer

**Affiliations:** 1 Department of Systems Biology and Bioinformatics, University of Rostock, Rostock, Germany; 2 Leibniz-Institute for Food Systems Biology at the Technical University Munich, Munich, Germany; 3 Division of Cell Biology and Physiology, CSIR-Indian Institute of Chemical Biology, Kolkata, India; 4 Academy of Innovative and Scientific Research, Ghaziabad, India; 5 Advanced Technology Development Centre, Indian Institute of Technology, Kharagpur, India; 6 Department of Computer Science & Engineering, Indian Institute of Technology, Kharagpur, India; 7 Stellenbosch Institute for Advanced Study (STIAS), Wallenberg Research Centre at Stellenbosch University, Stellenbosch, South Africa

**Keywords:** Type 2 diabetes, Epidemiology

## Abstract

**Background:**

Studies on Type-2 Diabetes Mellitus (T2DM) have revealed heterogeneous sub-populations in terms of underlying pathologies. However, the identification of sub-populations in epidemiological datasets remains unexplored. We here focus on the detection of T2DM clusters in epidemiological data, specifically analysing the National Family Health Survey-4 (NFHS-4) dataset from India containing a wide spectrum of features, including medical history, dietary and addiction habits, socio-economic and lifestyle patterns of 10,125 T2DM patients.

**Methods:**

Epidemiological data provide challenges for analysis due to the diverse types of features in it. In this case, applying the state-of-the-art dimension reduction tool UMAP conventionally was found to be ineffective for the NFHS-4 dataset, which contains diverse feature types. We implemented a distributed clustering workflow combining different similarity measure settings of UMAP, for clustering continuous, ordinal and nominal features separately. We integrated the reduced dimensions from each feature-type-distributed clustering to obtain interpretable and unbiased clustering of the data.

**Results:**

Our analysis reveals four significant clusters, with two of them comprising mainly of non-obese T2DM patients. These non-obese clusters have lower mean age and majorly comprises of rural residents. Surprisingly, one of the obese clusters had 90% of the T2DM patients practising a non-vegetarian diet though they did not show an increased intake of plant-based protein-rich foods.

**Conclusions:**

From a methodological perspective, we show that for diverse data types, frequent in epidemiological datasets, feature-type-distributed clustering using UMAP is effective as opposed to the conventional use of the UMAP algorithm. The application of UMAP-based clustering workflow for this type of dataset is novel in itself. Our findings demonstrate the presence of heterogeneity among Indian T2DM patients with regard to socio-demography and dietary patterns. From our analysis, we conclude that the existence of significant non-obese T2DM sub-populations characterized by younger age groups and economic disadvantage raises the need for different screening criteria for T2DM among rural Indian residents.

## Introduction

Type-2 diabetes mellitus (T2DM) is a multifactorial disease globally estimated to rise to 629 million cases by 2045 (see IDF Diabetes Atlas) [1, 2]. Though conceived as a homogeneous disease for long, several recent studies have found T2DM to be a mix of heterogeneous disease subtypes [3–5]. These studies have reported a varied pathophysiology underlying T2DM and thereby suggest the possibility of a personalised treatment for T2DM.

Besides obesity, other factors like age, sex, socio-economic status, place of residence (rural/urban), smoking habit, alcohol intake, food frequency, etc. are significantly associated with T2DM [6–13]. Several of these factors are modifiable in nature and hence are important in the management of T2DM [1]. However, modification of lifestyle-related factors varies and thereby leads to a differential degree of glycemic control among T2DM patients [14]. Glycaemic control and response to anti-diabetics have also been shown to be different among T2DM sub-groups [15]. To explore whether any particular pattern of patient sub-populations exists within the entire T2DM population based on socio-demographic and lifestyle factors, we used an unsupervised clustering approach on the largest and most comprehensive epidemiological dataset in India, the National Family Health Survey-4 (NFHS-4) dataset. Clusters were subsequently characterised to identify unique socio-demographic and lifestyle patterns associated with these sub-populations.

Epidemiological datasets provide a comprehensive set of information regarding socio-demography, lifestyle, addiction and co-morbidities. Variables containing such information are called *features* in the language of Machine Learning. In the T2DM-NFHS-4 dataset, there are 36 such features, containing information on each diabetes patient. Moreover, in our dataset, the features can be categorised into three types:

1. *Continuous features:* These are the features that can assume any numeric value from a continuous range. For example, the BMI of a patient is a continuous feature.
2. *Ordinal features:* These are the features that assume values from a discrete range, such that, there is a sense of order in the values assumed by the feature. For example, let us assume a feature ‘meat consumption by a patient’, assumes values ‘daily’, ‘weekly’ or ‘monthly’. Clearly, the range of the feature ‘meat consumption by a patient’ is discrete, since it can assume any one of the three values. Also, there is a sense of order in the values, indicating that daily meat consumption is the highest and monthly meat consumption is the lowest if we want to quantify meat consumption.
3. *Nominal features:* These are the features that assume values from a discrete range, such that, there is no sense of order in the values assumed by the feature. For example, let us assume a feature ‘religion of a patient’, assumes values ‘Hindus’, ‘Muslims’ or ‘Christians’. Clearly, the range of the feature ‘religion of a patient’ is discrete, since it can assume any one of the three values. But there is no sense of order in the possible values assumed by the features. Yet, this feature draws its importance from the fact that lifestyle patterns or diets vary largely among these religious groups.

Such diverse types of features in epidemiological data create challenges for the analysis. Conventional application of the state-of-the-art dimension reduction tool Uniform Manifold Approximation (UMAP) was found to be ineffective for the T2DM-NFHS-4 dataset. Continuous features, although smaller in numbers, had an overpowering effect on the distribution of clusters. To address this problem, we implemented a distributed clustering workflow, combining different similarity measure settings of UMAP, for clustering continuous, ordinal and nominal features separately. We integrated the reduced dimensions from each feature-type-distributed clustering to obtain interpretable and unbiased clustering of the data.

**Fig. 1:**
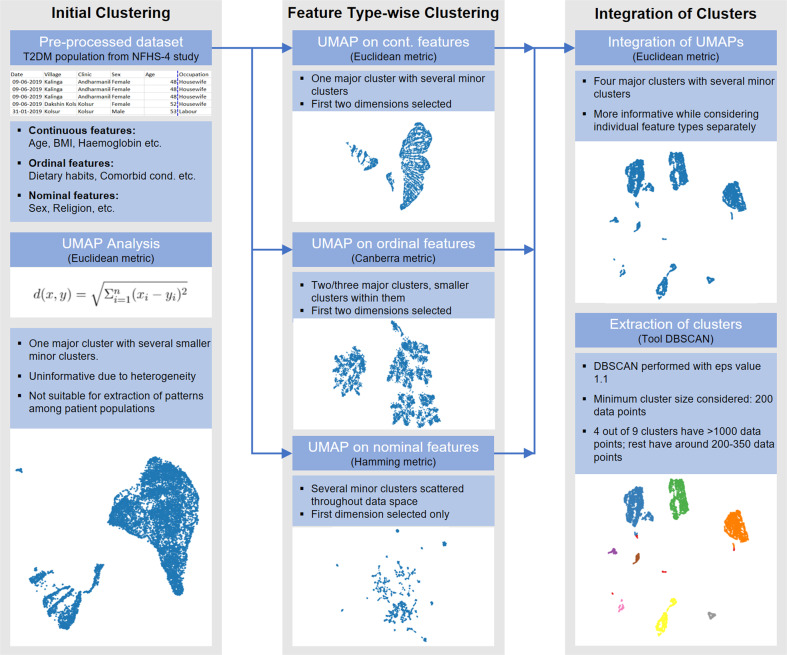
Workflow describing the analysis of the T2DM-NFHS-4 Dataset. A visualisation for the novel feature type-wise clustering paradigm used in the study, to account for the bias of UMAP towards over representation of continuous variables during dimension reduction.

The workflow realised for the present study (Fig. 1) involves the investigation of underlying socio-demographic patterns within patient sub-populations using unsupervised learning. Dimension reduction approaches are often used to reduce higher dimensional data to lower dimensions such that in the lower dimensional embedding of the data one can visualize underlying clusters within the data, that are not apparent in the higher dimensions [16]. Several such techniques have been developed over the last few decades. Until recently the dimension reduction technique t-Stochastic Neighbourhood Embedding (t-SNE) was a state-of-the-art algorithm in this field providing numerous applications in various fields [17–19]. t-SNE projects high dimensional data to a lower dimension while maintaining the underlying local manifold structure in a sense that, in a lower dimension t-SNE can cluster points, that are close enough in the latent high dimensional manifold [17].

With a rigorous mathematical foundation, considerably high speed and easy to use using scikit-learn API, UMAP has turned out to be one of the most popular choices among data scientists [20–22]. As opposed to t-SNE, UMAP uses a graph-based manifold approximation mechanism which contributes to the preservation of the global as well as social properties of the latent data manifold in a lower-dimensional representation of the data. Given some low-dimensional representation of the data, a similar process can be used to construct an equivalent topological representation. UMAP builds a graph considering customized neighbourhoods for every data point. This graph is a representation of the higher dimensional data manifold. The end result is a patchwork of low-dimensional representations of neighbourhoods that groups similar data points on a local scale while better preserving long-range topological connections to more distantly related data points [20, 22]. For the ability of UMAP to preserve the long-range topological connections along with the short-range topological connections and because of its high computational efficiency we choose UMAP for our unsupervised clustering approach. Moreover, UMAP allows a user to specify several similarity measures through the tuning of the metric parameter. This has been critical in our workflow since our data contains continuous and categorical features and choosing suitable similarity measures for continuous and categorical features is crucial for a meaningful and informative clustering [23].

## Methodology

### Source and description of the T2DM-NFHS-4 dataset

Data preparation and pre-processing are the key aspects of approaching a problem from a Machine Learning perspective. In this section, we provide the details on the pre-processing approach adopted to generate the T2DM-NFHS-4 dataset.

The NFHS-4 dataset was downloaded from The Demographic & Health Surveys (DHS) Program website. NFHS-4 is the fourth version of the national health survey conducted under the supervision of the Ministry of Health and Family Welfare, Government of India with the International Institute for Population Sciences (IIPS), Mumbai serving as the main nodal agency for all the surveys. The sampling procedure followed in NFHS-4 was stratified two-stage sampling covering all the 640 districts of India. The survey was successfully conducted with 601,509 households. In those interviewed households 112,122 men and 699,686 women could be successfully interviewed. Four survey questionnaires (Household Questionnaire, Woman’s Questionnaire, Man’s Questionnaire and Biomarker Questionnaire) were implemented in 17 local languages to collect information on basic demographic information, socio-economic parameters, family planning issues, nutritional status, health indicators, contact with community health workers, etc. The uniqueness of the NFHS-4 study was that it collected data on Diabetes status and performed a Random Blood Glucose for individuals (15–54 years) using a finger-stick blood specimen. As a result, the biomarker measurements and tests besides anthropometric measurements like anaemia testing, blood pressure measurement, blood glucose testing and HIV testing were included in the survey.

### Dataset preparation

For dataset preparation and cleaning, the three questionnaires were merged: Woman’s Questionnaire, Man’s Questionnaire and Biomarker Questionnaire. The first two contained information about background characteristics (location, age, sex, religion, social group, literacy, wealth status, etc.), nutritional practices, addictions and co-morbidities while the biomarker questionnaire contained information on height, weight, blood pressure and random blood glucose. A unique code was generated for all individuals in all the three questionnaires by appending the country code and phase, cluster number, household number and line number. The three datasets were joined by the unique code to prepare a single dataset of 810,971 individuals consisting of all men and women between 15–54 years of age. Pregnant women were next excluded to discard the possibility of Gestational Diabetes Mellitus. Individuals with missing diabetic and blood pressure status were also excluded. Variables known to be risk factors for DM (body mass index (BMI), age, place of residence, wealth index, smoking frequency, alcohol intake frequency, hypertension), socio-economic factors (sex, religion, social group, educational status), Dietary frequencies and haemoglobin level were selected for final analysis. BMI, age and haemoglobin level were taken as continuous variables and the rest as categorical variables. Outliers were removed separately for all the three continuous variables to obtain the final dataset with 610, 498 individuals (526, 678 females and 83, 820 males).

### Dataset pre-processing

We were interested in detecting significant T2DM sub-populations in the data and further sought to characterize these sub-populations based on the socio-demographic and co-morbid conditions. For this purpose, we extracted patients with a known history of diabetes from the dataset: a total of 10 125 patients. We considered a diverse collection of socio-demographic and co-morbid conditions as ‘features’ in our dataset. Qualitatively our features can be divided into several categories:

1. *Co-morbid conditions:* This class of features considers the co-morbid diseases among T2DM patients. We considered whether a T2DM patient had medical conditions such as asthma, thyroid disorder, heart disease, cancer, tuberculosis and hypertension. Thus, there were six features in this category. These features are binary in nature denoting whether a T2DM patient suffered from a given comorbidity or not.
2. *Food habits:* This class of features considered the food habits of T2DM patients. The features considered here were how frequently the patient took the food items: milk or curd, pulses or beans, dark leafy vegetables, fruits, eggs, fish, chicken, fried food and aerated drinks. Thus, there were nine features in this category. Features were categorical and ordinal in nature having four possible values: ‘daily’, ‘occasionally’, ‘weekly’ and ‘never’.
3. *Addiction history:* This class of features considered the addiction pattern of T2DM patients. There were two features in this class, both binary in nature encoding whether a patient is a smoker or whether a patient takes alcohol.
4. *Socio-demographic features:* These included features such as sex, age, wealth index, education level, religion and caste along with BMI and haemoglobin level of the patient. There were eight features in this category.
5. *Living conditions:* This class of features quantifies the living conditions of the patients. The features in this class considered whether a patient lives in a household possessing refrigerator, bicycle, motorbike, four-wheeler vehicle and livestock. Moreover, there were features denoting the type of residence, household structure, frequency of household members smoking inside the house, type of cooking fuel used, source of drinking water and time to reach the nearest drinking water source. Thus, there were eleven features belonging to this category.

For our study, 36 features or factors are considered to investigate significant patient populations among the diabetes patients into consideration. Note that there are both continuous and categorical features among these thirty-six features. Among the categorical features, there are both ordinal features and nominal features. Ordinal features have a sense of order among them, such as the features from the ‘food habits’ category as described before. The nominal features are categorical features with no sense of order such as the sex of a patient. Note that for our dataset the continuous features are: age, BMI, haemoglobin level and time to get to drinking water source, whereas the nominal features are: sex, religion, caste, household structure, type of place of residence, type of cooking fuel and source of drinking water. The rest of the features are ordinal features. The categorization of features into continuous, nominal and ordinal is of utmost importance in our clustering paradigm which we discuss in the section “Clustering paradigm using UMAP”.

### Identification of T2DM sub-populations using UMAP and DBSCAN

**Fig. 2:**
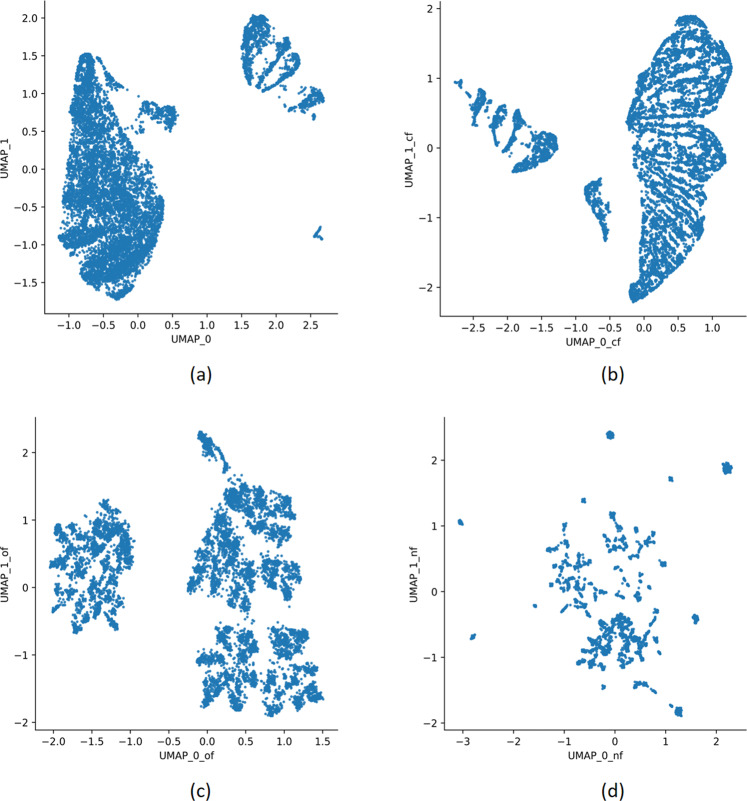
The low dimensional UMAP visualisations of data for several data types. **a** UMAP clusters for all the features with the Euclidean metric. **b** UMAP clusters for continuous features with Euclidean metric. **c** UMAP clusters for ordinal features with Canberra metric. **d** UMAP clusters for nominal features with Hamming metric.

From our detailed description of our dataset, we pointed out that our dataset has a variety of features including continuous and categorical features. Further, there are both ordinal and nominal features among the categorical features in our dataset. A simple UMAP on the entire dataset is depicted in Fig. 2a, revealing two broad clusters. For this clustering of UMAP parameters, n_neighbours have been chosen to be 30, whereas the metric parameter has been chosen to be Euclidean. However, we have a number of important nominal and ordinal categorical features whose effect would not be apparent from such a clustering. Moreover, the Euclidean distance does not always make sense on categorical features, especially if they are nominal in nature. For example, observe Fig. 2d, where we have used UMAP considering only the nominal features with metric parameter hamming (based on hamming distance). This reveals a completely different picture of the dataset, showing several small clusters. Our clustering paradigm is designed to optimise this effect and find a balance in the clustering where a particular type of feature does not have an overpowering effect on the clustering process.

### Clustering paradigm using UMAP

Our clustering paradigm applies UMAP separately on continuous, nominal and ordinal features separately. For each of these feature categories, we create a lower-dimensional embedding of the dataset. Finally, we integrate the lower-dimensional embeddings to extract clusters from them using the DBSCAN algorithm, a clustering algorithm used for extracting clusters from data based on data density. One advantage of this algorithm is that one does not need to specify the number of clusters beforehand. DBSCAN considers closely or densely located points, as clusters [24]. For UMAP, we use the same values for the parameters n_neighbours = 30 and min_distance = 0.1 for all the feature types.

- For the *continuous features,* we use the metric measure to be *Euclidean*. The Euclidean distance between two vectors is given by:
  1 $$d\left( {x,y} \right) = \sqrt {\mathop {\sum}\nolimits_{i = 1}^n {\left( {x_i - {\it{y}}_i} \right)^2} }$$
- For the *nominal features,* we use the metric measure to be *Hamming*. Hamming distance is defined as:
  2 $$d\left( {x,y} \right) = \mathop {\sum}\nolimits_{i = 1}^n {\delta \left( {x_i,y_i} \right)}$$
  where *δ*(*x*_*i*_, *y*_*i*_) = 1, if *x*_*i*_ = *y*_*i*_ and *δ*(*x*_*i*_, *y*_*i*_) = 0 otherwise. Recall that nominal features are also a type of categorical features that do not have a sense of order associated with them. For such features, Hamming distance is widely used as a similarity measure between data points [23].
- For the *ordinal features,* we use the metric measure to be *Canberra*. It is a weighted version of the Manhattan measure. The Canberra distance is given by:

3 $$d\left( {x,y} \right) = \sqrt {\mathop {\sum}\nolimits_{i = 1}^n {\frac{{\left| {x_i - y_i} \right|}}{{\left| {x_i} \right| + \left| {y_i} \right|}}} }$$

Ordinal features are also a type of categorical feature. However, the Hamming metric cannot capture the inherent ordered relationships and statistic information from categorical values [23]. We thus tried using UMAP for several metric measures and noticed that the Canberra distance measure retains a high variance in the lower dimensions. Thus we chose the Canberra distance measure as a similarity metric for ordinal features.

For the categorical and ordinal features, we thus produce a two-dimensional representation of each data point by taking into consideration the first two UMAP coordinates. For the nominal features, we consider we produce a one-dimensional representation since the data points are too scattered in this case as shown in Fig. 2d and thus can lead to too many clusters. Thus, we reduce every data point into a five-dimension representation, two for each of the continuous and ordinal features and one for the nominal features. Finally, we look for clusters in the five-dimensional representation using DBSCAN (eps = 1, min_points = 200). After selecting the final clusters, we characterized them by summarizing all the 36 variables separately for each cluster. The continuous variables were summarized as their mean and the standard error of the mean. The categorical variables were summarized as their frequency distribution and the proportion of each value within each cluster.

### Extraction of T2DM sub-populations using DBSCAN

**Fig. 3:**
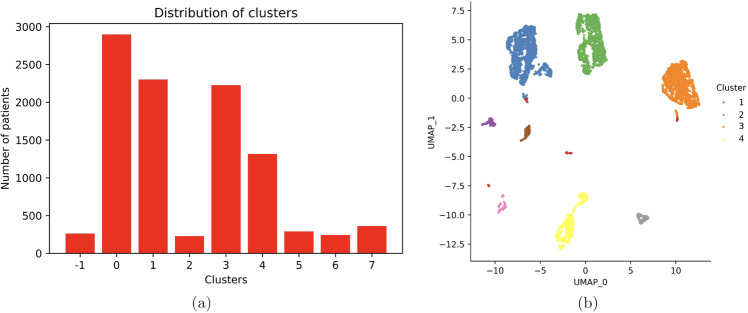
The information on clusters detected in the data. **a** Distribution of clusters detected by DBSCAN on the five-dimensional reduced representation of the data. **b** UMAP clusters for five-dimensional reduced representation of the data annotated by the DBSCAN generated clusters.

Using our clustering paradigm described before, we can detect seven sub-populations among the patients where 261 patients are considered as outliers. We show the distribution of clusters in Fig. 3a. We further perform a UMAP on the five-dimensional reduced representation of our data to visualize the clusters detected by DBSCAN. For this, we label the data points using the DBSCAN clustering labels and colour code them in the UMAP representation of the five-dimensional reduced data as shown in Fig. 3b. This provides validation to the fact the clustering done by DBSCAN makes sense. Note that, from our clusters, we can detect four significant patient sub-populations containing 2898, 2301, 2226 and 1315 data points.

## Results

### Characterization of clusters

#### Age and BMI both were found to be lower in Cluster 2 and Cluster 4

Age and obesity are the most important risk factors for T2DM. However, we found a heterogeneity in both these variables across all the clusters. Interestingly, the mean age and BMI both were lower in Cluster 2 (Age: 38.3 ± 0.19 years, BMI: 23.9 ± 0.1) and Cluster 4 (Age: 37.9 ± 0.26 years, BMI: 23.6 ± 0.13) compared to Cluster 1 (Age: 41.3 ± 0.14 years, BMI: 26.7 ± 0.09) and Cluster 3 (Age: 39.9 ± 0.18 years, BMI: 26 ± 0.11). However, the distribution of males and females has been found to be similar across all the clusters.

#### Higher proportion of rural residents and lower proportion of richest wealth quintile in Clusters 2 and 4

The proportion of rural residents was found to be high in Cluster 2 (69.4% were Rural residents) and Cluster 4 (72.02% were Rural residents) compared to the other clusters (31.3% in Cluster 1 and 49.19% in Cluster 3). Surprisingly, only 4.3% of the people in Cluster 2 and 8.37% in Cluster 4 belonged to the richest quintile of the Wealth Index category whereas 64.04% in Cluster 1 and 54.9% in Cluster 3 belonged to the same.

#### Frequency of co-morbid conditions was similar across all the clusters

Co-morbid conditions included a history of asthma, thyroid disease, heart disease, cancer, history of tuberculosis, haemoglobin level and hypertension. Though the distribution of disease conditions shows minor variation across the clusters (Table 1), the trend is almost similar in all the clusters.

**Table 1:** Detailed cluster-specific analysis for all numerical and categorical variables.

**Identified clusters**	**Cluster 1**	**Cluster 2**	**Cluster 3**	**Cluster 4**
Cluster size (N)	2898	2301	2226	1315
Cont. variables (Mean ± SE)				
Age (years)	41.3 ± 0.14	38.3 ± 0.19	39.9 ± 0.18	37.9 ± 0.26
Body mass index (kg/m^2^)	26.7 ± 0.09	23.9 ± 0.1	26 ± 0.11	23.6 ± 0.13
Haemoglobin (gm/dl)	12.5 ± 0.04	12.3 ± 0.04	12.1 ± 0.04	12.3 ± 0.06
Time to the water source (min)	0.1 ± 0.01	0.02 ± 0.01	0.09 ± 0.01	18.6 ± 0.39
Cat.variables				
Value for cat. variables				
Sex				
Male	558 (19.25)	457 (19.86)	270 (12.13)	323 (24.56)
Female	2340 (80.75)	1844 (80.14)	1956 (87.87)	992 (75.44)
History of asthma				
No	2737 (94.44)	2064 (89.7)	1999 (89.8)	1121 (85.25)
Yes	161 (5.56)	237 (10.3)	227 (10.2)	194 (14.75)
History of thyroid disorder				
No	2636 (90.96)	2135 (92.79)	1992 (89.49)	1196 (90.95)
Yes	262 (9.04)	166 (7.21)	234 (10.51)	119 (9.05)
History of heart disease				
No	2729 (94.17)	2107 (91.57)	1996 (89.67)	1174 (89.28)
Yes	169 (5.83)	194 (8.43)	230 (10.33)	141 (10.72)
History of cancer				
No	2876 (99.24)	2272 (98.74)	2161 (97.08)	1246 (94.75)
Yes	22 (0.76)	29 (1.26)	65 (2.92)	69 (5.25)
Ever suffered from TB				
No	2890 (99.72)	2287 (99.39)	2218 (99.64)	1305 (99.24)
Yes	8 (0.28)	14 (0.61)	8 (0.36)	10 (0.76)
Milk/curd intake freq.				
Never	201 (6.94)	183 (7.95)	110 (4.94)	123 (9.35)
Weekly	461 (15.91)	551 (23.95)	293 (13.16)	405 (30.8)
Occasionally	611 (21.08)	669 (29.07)	447 (20.08)	291 (22.13)
Daily	1625 (56.07)	898 (39.03)	1376 (61.81)	496 (37.72)
Pulses/beans intake freq.				
Never	13 (0.45)	17 (0.74)	18 (0.81)	9 (0.68)
Weekly	255 (8.8)	248 (10.78)	152 (6.83)	198 (15.06)
Occasionally	1263 (43.58)	937 (40.72)	936 (42.05)	574 (43.65)
Daily	1367 (47.17)	1099 (47.76)	1120 (50.31)	534 (40.61)
Green vegetables intake freq.				
Never	7 (0.24)	12 (0.52)	10 (0.45)	9 (0.68)
Weekly	324 (11.18)	259 (11.26)	279 (12.53)	142 (10.8)
Occasionally	1000 (34.51)	796 (34.59)	792 (35.58)	483 (36.73)
Daily	1567 (54.07)	1234 (53.63)	1145 (51.44)	681 (51.79)
Fruit intake freq.				
Never	50 (1.73)	65 (2.82)	74 (3.32)	41 (3.12)
Weekly	897 (30.95)	1148 (49.89)	872 (39.17)	750 (57.03)
Occasionally	1203 (41.51)	818 (35.55)	810 (36.39)	386 (29.35)
Daily	748 (25.81)	270 (11.73)	470 (21.11)	138 (10.49)
Egg intake freq.				
Never	97 (3.35)	85 (3.69)	1983 (89.08)	41 (3.12)
Weekly	1005 (34.68)	963 (41.85)	153 (6.87)	520 (39.54)
Occasionally	1537 (53.04)	1100 (47.81)	80 (3.59)	678 (51.56)
Daily	259 (8.94)	153 (6.65)	10 (0.45)	76 (5.78)
Fish intake freq.				
Never	222 (7.66)	106 (4.61)	2162 (97.12)	83 (6.31)
Weekly	994 (34.3)	1006 (43.72)	35 (1.57)	593 (45.1)
Occasionally	1210 (41.75)	987 (42.89)	20 (0.9)	563 (42.81)
Daily	472 (16.29)	202 (8.78)	9 (0.4)	76 (5.78)
Chicken/meat intake freq.				
Never	53 (1.83)	58 (2.52)	2175 (97.71)	33 (2.51)
Weekly	1274 (43.96)	1150 (49.98)	32 (1.44)	640 (48.67)
Occasionally	1475 (50.9)	1032 (44.85)	18 (0.81)	612 (46.54)
Daily	96 (3.31)	61 (2.65)	1 (0.04)	30 (2.28)
Fried food intake freq.				
Never	179 (6.18)	161 (7)	276 (12.4)	95 (7.22)
Weekly	1275 (44)	988 (42.94)	1114 (50.04)	631 (47.98)
Occasionally	1071 (36.96)	849 (36.9)	715 (32.12)	408 (31.03)
Daily	373 (12.87)	303 (13.17)	121 (5.44)	181 (13.76)
Aerated drink intake freq.				
Never	512 (17.67)	475 (20.64)	409 (18.37)	262 (19.92)
Weekly	1579 (54.49)	1258 (54.67)	1200 (53.91)	744 (56.58)
Occasionally	597 (20.6)	449 (19.51)	497 (22.33)	236 (17.95)
Daily	210 (7.25)	119 (5.17)	120 (5.39)	73 (5.55)
Alcoholic				
No	2627 (90.65)	2027 (88.09)	2171 (97.53)	1127 (85.7)
Yes	271 (9.35)	274 (11.91)	55 (2.47)	188 (14.3)
Smoker				
No	2770 (95.58)	2192 (95.26)	2197 (98.7)	1234 (93.84)
Yes	128 (4.42)	109 (4.74)	29 (1.3)	81 (6.16)
Indoor smoking freq.				
Never	1849 (63.8)	1138 (49.46)	1429 (64.2)	690 (52.47)
Weekly	222 (7.66)	264 (11.47)	176 (7.91)	129 (9.81)
Less than monthly	72 (2.48)	72 (3.13)	71 (3.19)	33 (2.51)
Monthly	78 (2.69)	72 (3.13)	68 (3.05)	36 (2.74)
Daily	677 (23.36)	755 (32.81)	482 (21.65)	427 (32.47)
Residence				
Urban	1991 (68.7)	704 (30.6)	1131 (50.81)	368 (27.98)
Rural	907 (31.3)	1597 (69.4)	1095 (49.19)	947 (72.02)
Wealth index				
Poorest	1 (0.03)	287 (12.47)	82 (3.68)	301 (22.89)
Poorer	8 (0.28)	519 (22.56)	154 (6.92)	285 (21.67)
Middle	151 (5.21)	698 (30.33)	245 (11.01)	339 (25.78)
Richer	882 (30.43)	698 (30.33)	523 (23.5)	280 (21.29)
Richest	1856 (64.04)	99 (4.3)	1222 (54.9)	110 (8.37)
Highest education level				
No education	388 (13.39)	758 (32.94)	416 (18.69)	472 (35.89)
Primary level	347 (11.97)	373 (16.21)	303 (13.61)	240 (18.25)
Secondary level	1641 (56.63)	1006 (43.72)	1106 (49.69)	530 (40.3)
Higher level	522 (18.01)	164 (7.13)	401 (18.01)	73 (5.55)
Religion				
Hindu	1822 (62.87)	1544 (67.1)	1947 (87.47)	975 (74.14)
Muslim	627 (21.64)	472 (20.51)	46 (2.07)	210 (15.97)
Christian	313 (10.8)	210 (9.13)	13 (0.58)	97 (7.38)
Others	136 (4.69)	75 (3.26)	220 (9.88)	33 (2.51)
Caste/tribe				
OBC	1331 (45.93)	871 (37.85)	805 (36.16)	472 (35.89)
SC	384 (13.25)	517 (22.47)	328 (14.73)	343 (26.08)
ST	303 (10.46)	385 (16.73)	86 (3.86)	258 (19.62)
General	880 (30.37)	528 (22.95)	1007 (45.24)	242 (18.4)
Hypertensive				
No	1594 (55)	1443 (62.71)	1281 (57.55)	849 (64.56)
Yes	1304 (45)	858 (37.29)	945 (42.45)	466 (35.44)
Possess refrigerator				
No	131 (4.52)	2296 (99.78)	762 (34.23)	989 (75.21)
Yes	2767 (95.48)	5 (0.22)	1464 (65.77)	326 (24.79)
Possess bicycle				
No	1503 (51.86)	1055 (45.85)	1013 (45.51)	617 (46.92)
Yes	1395 (48.14)	1246 (54.15)	1213 (54.49)	698 (53.08)
Possess motorbike				
No	825 (28.47)	1590 (69.1)	734 (32.97)	884 (67.22)
Yes	2073 (71.53)	711 (30.9)	1492 (67.03)	431 (32.78)
Possess car/truck				
No	2217 (76.5)	2226 (96.74)	1840 (82.66)	1273 (96.81)
Yes	681 (23.5)	75 (3.26)	386 (17.34)	42 (3.19)
Cooking fuel used				
Other	1 (0.03)	4 (0.17)	0 (0)	1 (0.08)
Plant based	354 (12.22)	1018 (44.24)	437 (19.63)	723 (54.98)
Livestock based	47 (1.62)	297 (12.91)	211 (9.48)	104 (7.91)
Gas/oil	2460 (84.89)	965 (41.94)	1562 (70.17)	476 (36.2)
Electricity	36 (1.24)	17 (0.74)	16 (0.72)	11 (0.84)
Household structure				
Non-nuclear	1310 (45.2)	1016 (44.15)	1120 (50.31)	564 (42.89)
Nuclear	1588 (54.8)	1285 (55.85)	1106 (49.69)	751 (57.11)
Possess livestock				
No	2226 (76.81)	1155 (50.2)	1474 (66.22)	646 (49.13)
Yes	672 (23.19)	1146 (49.8)	752 (33.78)	669 (50.87)
Drinking water source				
Unprotected sources	76 (2.62)	146 (6.35)	44 (1.98)	204 (15.51)
Protected sources	739 (25.5)	998 (43.37)	686 (30.82)	522 (39.7)
Community service	1991 (68.7)	1112 (48.33)	1448 (65.05)	508 (38.63)
Bottled water	86 (2.97)	43 (1.87)	46 (2.07)	77 (5.86)
Other	6 (0.21)	2 (0.09)	2 (0.09)	4 (0.3)

#### Lifestyle patterns show evidences of a lower quality of life for patient sub-populations in Clusters 2 and 4

Our analysis reveals several other factors that support the fact that T2DM sub-populations from Cluster 2 and Cluster 4 have a considerably lower quality of life.

1. We observe that only 0.22% and 24.79% of patients belonging to Cluster 2 and Cluster 4, respectively, possess a refrigerator compared to 95.48% and 65.77% of patients belonging to Cluster 1 and Cluster 3, respectively.
2. Only 30.9% and 32.78% of patients belonging to Cluster 2 and Cluster 4 respectively possess a motorbike compared to 71.53% and 67.03% of patients belonging to Cluster 1 and Cluster 3, respectively.
3. Only 3.26% and 3.19% of patients belonging to Cluster 2 and Cluster 4 respectively possess a car/truck compared to 23.5% and 17.34% of patients belonging to Cluster 1 and Cluster 3, respectively.
4. 44.24% and 54.98% of patients belonging to Cluster 2 and Cluster 4 respectively, use plant-based cooking fuel, which is relatively cheap, compared to 12.22% and 19.63% of patients belonging to Cluster 1 and Cluster 3 respectively. Moreover, only 41.94% and 36.2% of patients belonging to Cluster 2 and Cluster 4, respectively use gas/oil-based cooking fuel, which is relatively expensive, compared to 84.89 and 70.17% of patients belonging to Cluster 1 and Cluster 3, respectively.
5. 6.35% and 15.51% of patients belonging to Cluster 2 and Cluster 4 respectively, drink water from unprotected sources, compared to 2.62% and 1.98% of patients belonging to Cluster 1 and Cluster 3, respectively.

#### Intake of non-vegetarian foods is invariably low in Cluster 3

Around 90% of the population in Cluster 3 had no intake of Egg (89.08%), fish (97.12%), chicken or meat (97.71%) whereas only less than 10% of the population in all the other 3 clusters had no intake of these non-vegetarian foods (Table 1). Though the Cluster 3 population had the highest daily intake of milk/curd (61.81%) and pulses/beans (50.31%) compared to the other clusters, other clusters also had an almost similar proportion of people taking milk/curd and pulses/beans daily. Intake of other foods like dark leafy vegetables, fruits, fried foods and aerated drinks showed similar distribution across all the clusters.

## Discussion

### Rationale of the workflow in clustering epidemiological data

The clustering workflow used arises from some important observations that we will discuss here. To begin with, we have a population of 10,125 T2DM patients with a diverse ensemble of features accounting for information on medical history, dietary and addiction habits, socio-economic and lifestyle patterns. Moreover, the features in the considered dataset are also diverse in terms of data types. We have a total of 36 features, out of which 4 are continuous features, 7 nominal features and 25 ordinal features, all of equal importance by assumption.

The aim is to find significant sub-populations in our data such that the identified sub-populations are interpretable in terms of the considered features. Note here that, by significant sub-populations, we mean a sub-population consisting of at least 10 percent of the total population. If there exist such sub-populations and we can explain the sub-populations in terms of the considered features, we can argue that these patterns exist in a significant number of patients.

We have already argued in favour of using UMAP for our unsupervised approach to find clusters in the data. However, we observed that applying UMAP algorithm conventionally using the Euclidean similarity metric on our entire dataset with 36 features turns out to be ineffective. The reason is, in this case, the continuous features have an overpowering effect over the other feature types in determining the distribution of clusters. This can be observed in Fig. 2a, b. Note that Fig. 2a shows UMAP clustering with all 36 features and 2(b) shows UMAP clustering with only four continuous features. Note that, there is a similarity in the clustering distribution of these figures, each containing one major cluster and seven small minor clusters. We observed that this is because of the fact that UMAP, when applied to all 36 features of the dataset using the Euclidean similarity measure is largely biased towards finding similarity among data points only in terms of the continuous features. Given that we have only four continuous features out of 36, this poses a problem as the diverse information present in the dataset in the form of the ordinal and nominal features is largely ignored.

To solve this problem, the clustering of continuous, ordinal and nominal features was treated separately by using different similarity matrices for them, giving rise to our clustering paradigm. We argued on our choice of similarity measures in Section “Clustering paradigm using UMAP”. This generates for each feature type a data representation of lower dimension shown in Fig. 2b–d. We finally integrated these lower dimension data representations by taking two-dimensional representations for continuous and ordinal features and a one-dimensional representation (the one consisting of the most variance) for nominal features. The reason behind considering one-dimensional representation for nominal features is that using Hamming metrics for such data results in retaining a lot of variance in the data resulting in multiple clusters as we observe in Fig. 2d. Considering a two-dimensional representation for this data while integrating these lower dimension data representations carry forward this variance and result in multiple small clusters in the final clustering distribution, which contradicts our aim of finding significantly large sub-populations (of at least 10 percent of the total population).

Finally, the integration is done by applying UMAP on the five-dimensional reduced representation of the dataset using the Euclidean similarity measure (shown in Fig. 3b). Note here that, in our final clusters, we can observe patterns in all of the continuous, ordinal and nominal data types. For example, in Cluster 4 the continuous feature ‘Time to Water source (min)’ shows very high values compared to other clusters. In Clusters 1 and 3, the nominal feature ‘Cooking fuel used’ shows a higher percentage for Gas/Oil users while in Clusters 2 and 4 the same feature shows a higher percentage for plant-based fuel users. In Cluster 3, the ordinal feature ‘Fish intake frequency’ shows 97% of people to be never consuming fish. Thus, we infer that our clustering paradigm enables us to find significant sub-populations while keeping the clustering distribution unbiased, that is no feature type continuous, ordinal and nominal has an overpowering effect on the other.

### Significance of T2DM clusters

T2DM was identified as a homogeneous disease with Insulin Resistance followed by *β*-cell dysfunction being the underlying pathology. However, recent studies have explored and found T2DM to be a heterogeneous entity with the relative contribution of Insulin Resistance and *β*-cell dysfunction to differ across T2DM clusters [3]. These studies were performed on clinical and biochemical data with variables having uniform data types. On the other hand, our clustering approach takes into account the diverse data types obtained from an epidemiological dataset and discovers clusters among the T2DM population. Interestingly, two of the four clusters obtained in our study belonged to the non-obese T2DM phenotype where the mean BMI was below 25. These two non-obese clusters also had lower mean age compared to the other clusters. Both these non-obese clusters had a larger proportion of rural residents and a lower proportion of people belonging to the highest wealth quintile concluding to the fact that a large majority of T2DM people from rural India have lower BMI and are younger in age. The T2DM patient sub-population belonging to these clusters has a relatively lower quality of life judging by analysis of the lifestyle pattern-based features. The non-obese phenotype of T2DM has been increasingly reported over the last two decades raising concern about the uniqueness of its underlying pathophysiology with a greater contribution of *β*-cell dysfunction compared to Insulin Resistance [25–28]. This non-obese T2DM phenotype has been found among Asians and studies depicting and investigating its similarities and differences have been in place. Studies have concluded that T2DM occurs among Asians at a lower BMI cut-off and also at a younger age [29, 30]. This finding of two non-obese clusters with lower mean age provides confirmation to this. Among the studies aimed to identify T2DM subtypes, the two subtypes severe insulin deficient diabetes (SIDD) and mild-age-related diabetes (MARD) were found to be common [3, 4]. Both cluster 2 and cluster 4 in our study seem to have similarities to the SIDD subtype though the reasons behind obtaining two different epidemiological clusters within the SIDD subtype need further investigation. As our dataset had patients below 49 years of age, we couldn’t obtain any cluster that may be compared to the mild-age-related diabetes (MARD) subgroup. The remaining two clusters in our study, cluster 1 and cluster 3 are both obese groups and therefore may be the epidemiological counterparts of either mild obesity-related diabetes (MOD) or severe insulin-resistant diabetes (SIRD). Hence, the T2DM clusters obtained from epidemiological data provide further strength to the clinical T2DM subtypes and raise the need to further investigate the epidemiological risk factors of T2DM subtypes.

Though non-obese T2DM is being considered a unique phenotype, epidemiological studies for identifying high-risk population groups still remain undone. This is especially important for many Asian countries where over half of the T2DM population is of non-obese phenotype [25]. This analysis, reporting an increased presence of Rural residents in both the non-obese T2DM clusters, calls for a modification in BMI and Age cut-off for T2DM screening among rural residents. However, identification of risk factors for T2DM specific to the rural population needs to be done. Representation of people from the highest wealth quintile was much lower in both the non-obese T2DM clusters. T2DM is a multifactorial disease requiring strict compliance to lifestyle modification, proper diet and antidiabetic therapy. Non-obese T2DM clusters with reduced representation from the highest wealth quintile suggest the possibility of unequal access to care for non-obese T2DM people thereby generating the need for a more equitable healthcare policy in terms of prevention and therapy.

On the other hand, both the obese T2DM clusters had higher ages and more urban residents. The proportion of people from the highest wealth quintile was higher in both the obese clusters. Interestingly one of the obese clusters (Cluster 3) had invariably a low intake of non-vegetarian foods (egg, fish, chicken and meat) pointing out the fact this T2DM cluster comprised vegetarian people mainly. Dietary requirements in diagnosed T2DM patients involve a reduced amount of carbohydrates and fats with an increased amount of protein-rich foods [31]. Animal products, being rich sources of dietary protein, need to be included in the diet. One of the obese T2DM clusters with a strict vegetarian dietary pattern suggests the need to design proper dietary guidelines for this group.

As already mentioned, T2DM is a multifactorial disease with socio-economic inequality suggested to play an important role in the pathology and management of the disease [32]. Studies have identified socio-economic inequalities and allostatic load to associate with T2DM [33]. The negative effect of social stress, uncertainty and poor nutrition [34] seems to be more relevant for clusters 2 and 4 in this study where individuals majorly belong to a weaker socio-economic class. Though this study doesn’t have the data to investigate this association, the possibility of obtaining T2DM subtypes based on the allostatic load seems to be a promising area of diabetes research. Designing a customized healthy diet and lifestyle plan for certain T2DM subtypes with a view to reducing the allostatic load may emerge as an important strategy in T2DM management.

## Conclusions

From a data science perspective, this analysis addresses the issue of diverse data types. We have shown that for such data conventional application of dimension reduction approaches might not be fruitful. We develop a workflow that contributes to finding meaningful and interpretable clusters such that the distribution of clusters is not biased by the data types.

The existence of a significant non-obese T2DM patient sub-population belonging to a younger age group and having larger proportions of rural residents raise a lower quality of life, indicating the need for different screening criteria for T2DM among rural Indian residents. The obese T2DM cluster with around 90% of people sticking to the non-vegetarian diet calls for the need for dietary guidelines for T2DM patients having a non-vegetarian dietary pattern.

## Data Availability

We support the idea of transparency and reproducibility of research. Therefore, all data relevant to this work are made publicly available on a GitHub repository.

## Code availability

The Python code (in the form of a Jupyer notebook) for the implementation of our workflow is also provided publicly in GitHub.
